# Navigating Dementia and Delirium: Balancing Identity and Interests in Advance Directives

**DOI:** 10.1111/nup.70016

**Published:** 2025-01-27

**Authors:** M. Rutenkröger

**Affiliations:** ^1^ Department of Medical Psychology University Medical Center Hamburg‐Eppendorf Hamburg Germany; ^2^ Uehiro Oxford Institute University of Oxford Oxford UK

**Keywords:** advance directive, delirium, dementia, identity, moral authority, self‐ownership

## Abstract

The moral authority of advance directives (ADs) in the context of persons living with dementia (PLWD) has sparked a multifaceted debate, encompassing concerns such as authenticity and the appropriate involvement of caregivers. Dresser critiques ADs based on Parfit's account of numeric personal identity, using the often‐discussed case of a PLWD called Margo. She claims that dementia leads to a new manifestation of Margo emerging, which then contracts pneumonia. Dworkin proposes that critical interests, concerning one's higher moral values, trump experiential interests (things or activities one enjoys because they are pleasurable). Dresser argues that Margo's current experiential interests override her self's critical ones, as they contribute significantly to her quality of life (QoL). To render the argument more realistic, I introduce a variation in which Margo develops delirium, a common and severe comorbidity in PLWD. I argue that delirium could precipitate a sudden decline in experiential interests and, consequently, a deterioration in QoL. Given the uncertain trajectory of Margo's illness, I contend that her competent self's critical interests, as reflected in her AD, along with her right to self‐ownership, should take precedence over current experiential interests. Thus, the AD possesses moral authority. However, it is imperative for healthcare professionals to offer consultations for PLWD, facilitating an understanding of ADs and enabling a shared decision‐making process. Such consultations are essential for honouring the autonomy and dignity of PLWD, ensuring that their values and preferences guide ethical decision‐making amidst the complexities of dementia care.

## 1 Introduction

About 1.8 million people in Germany are currently living with dementia, Alzheimer's disease being the most prevalent form (German Alzheimer Society, e.V 2023). The disease is characterised by a progressive decline in cognitive abilities, such as memory and executive functions, but also behavioural and psychological symptoms, for example, agitation, anxiety, irritability, depression, disinhibition, delusions and sleep or appetite changes (Cerejeira, Lagarto, and Mukaetova‐Ladinska 2012). Because of the loss of cognitive functioning, patients are usually considered legally incapable, as they can no longer predict the consequences of their actions (Kaufmann 2015). Persons living with dementia (PLWD) are encouraged to express their preferences concerning life‐sustaining treatment and palliative care in an advance directive (AD)^1^ while still competent (Fischer et al. 2023).

However, the moral authority of ADs in the case of dementia has been questioned for several reasons, for example, authenticity (Vollmann 2001) or inappropriate involvement of caregivers during the preparation phase (Fischer et al. 2023). The most challenging argument seems to be the personal identity problem. Parfit (1984) inquiry into personal identity pertains to the question of what makes a person the same over time, and how our sense of identity is related to the continuity and changes of our physical and psychological characteristics. In relation to ADs, the personal identity problem questions whether the person before and after the onset of dementia should be regarded the same. This problem is often discussed using Dworkin's example of a ‘happy and content’ PLWD called Margo (Dworkin 1993), who in her AD refused life‐sustaining measures in case of dementia when she was fully competent, before receiving an Alzheimer's diagnoses.

Discussing the moral authority of ADs, Dworkin defines critical interests as ‘interests that it does make their life genuinely better to satisfy, interests they would be mistaken, and genuinely worse off, if they did not recognise,’ such as establishing close friendships or raising children; but also the desire to die with dignity^2^ and avoid undue suffering (Dworkin 1993, 201). On the other hand, he describes experiential interests as things or activities ‘we all do because we like the experience of doing them: playing softball (…), or seeing Casablanca for the twelfth time’ (Dworkin 1993, 200). He argues that the latter highly depend on the fact that one finds them pleasurable, making them highly individual. While he acknowledges that experiential interests are essential to a good life, he assigns greater moral weight to critical interests.

In Margo's case, Dworkin defends that her AD should be followed as it reflects her critical interests, that is, her values in coherence with her prior personality and life goals and suggests that narrative coherence with a person's former life decisions is a valid justification for pursuing a PLWD's precedent autonomy (Dworkin 1993). Dresser, on the other hand, argues that Margo's current experiential interests should override pre‐dementia Margo's critical interests as her quality of life (QoL) is comparatively good, stating that ‘(h)appy and contented Margo will experience clear harm from the decision that purports to advance the critical interests she no longer cares about’ (Dresser 1995, 36). In contrast to Dworkin, she suggests that most people do not distinguish between experiential and critical interests, but instead adapt to social and environmental changes in their daily lives (Dresser 1995, 36). Relying on Parfit's premise of psychological continuity^3^ for personal identity, Dresser argues that pre‐dementia Margo (Margo 1) and Margo with advanced dementia (Margo 2) are two different persons and that, therefore, Margo 1's AD lacks moral authority (Dresser 1995, 38). This is so because ADs ought only to apply to the person who drafted it.

In this essay, I will argue that personal identity does not reduce the moral authority of ADs in the way Dresser claims. I will suggest that Margo's case of a happy and content person with advanced dementia is somewhat atypical and that dementia may create several numerical identities of a person within one body due to an ongoing cognitive decline as well as comorbidities, such as delirium. As PLWD experience a progressive decline in cognitive capacity and associated symptoms such as delusions and unsafe behaviours, this may lead to an unequal balance between pleasure and pain, resulting in a decrease in experiential interests and a poorer QoL. I conclude that as dementia's complications contribute to an uncertain course of Margo's illness, her critical interests as reflected by her AD and her right to self‐ownership over her body, ought to have moral authority over new numerical identities. I then discuss possible objections.

## 2 Different Views on Critical Interests and QoL

The most persuasive component of Dresser's argument in terms of Margo 2's state of advanced dementia is her comparatively good QoL. She enjoys simple activities and seems content while not being in pain or distress; then, she contracts pneumonia and needs antibiotics to prevent her from dying. Dresser argues that Margo 2's experiential interests are what matters as they significantly contribute to her QoL and that Margo 1's former critical interests no longer reflect that (Dresser 1995). Contrary, Dworkin implies that Margo 1's critical interests persist even if Margo 2 does not appreciate them,^4^ and still hold more moral weight than her current experiential interests. Dworkin's view seems to be consistent with the idea of surviving interests (Buchanan and Brock 1991). This concept suggests that interests can persist even after their subject is lost, as long as the object of the preference (i.e., Margo's body) remains. I will limit my argument to only consider critical interests regarding one's body.

The concept of surviving critical interests does not appear to undermine Dresser's position. Dresser does not reject that Margo 1's critical interests may persist beyond her ‘death’, she instead argues that they are irrelevant to Margo 2's life as these are no longer her own since Margo 1 and 2 are different numerical persons (Dresser 1995). The discrepancy between Dworkin's and Dresser's positions is their differing assessment of the moral weight of critical interests and their meaning in terms of QoL.

A third position on critical and experiential interests and QoL is the hedonistic view. Hedonism places a strong emphasis on maximising pleasure and happiness as the ultimate goal for an individual's well‐being. According to hedonism, the pursuit of experiential pleasures takes precedence over other considerations, such as critical interests. In this context, experiential pleasures refer to the immediate and tangible sources of enjoyment and satisfaction that individuals can experience in their daily lives. This may include activities like indulging in pleasurable sensory experiences, pursuing leisure and entertainment, or engaging in activities that provide a sense of joy and fulfilment. On the other hand, critical interests typically involve intellectual, moral, or ethical pursuits that may not necessarily contribute directly to immediate happiness or pleasure. Examples of critical interests may include engaging in philosophical discussions, pursuing justice, or advocating for certain moral principles. The hedonistic perspective argues that since critical interests do not directly contribute to immediate happiness or pleasure, they hold no moral weight in the overall assessment of an individual's well‐being. In other words, for a hedonist, the value of an activity or interest is determined by its capacity to enhance immediate happiness and pleasure. Thus, experiential pleasures are to be prioritised to maximise the overall well‐being of a person.

So far, I have suggested that it seems not important whether one assumes that critical interests outlive Margo's death but what moral weight one attaches to these interests. In cases of advanced dementia, we now have to question whether or not ADs based on critical interests have moral authority over new numerical identities. For doing so, it is important to consider how realistic Margo's case of a happy and contented person with advanced dementia is.

## 3 Advanced Dementia and Associated Complications

Given current evidence, QoL drastically decreases and psychological burden increases with the progression of Alzheimer's disease (Froelich et al. 2021). Generally speaking, Margo 2's situation of being happy and contented while being in an advanced state of dementia can be considered somewhat atypical. Research indicates that PLWD commonly experience coexisting medical ailments and associated complications. The presence of dementia may pose challenges to the clinical management of other conditions and further impede a patient's capacity to cope with other illnesses (Bunn et al. 2014).

In the often‐discussed example, Margo contracts pneumonia which is considered a severe disease in the elderly. In fact, a study by Degerskär and Englund (2020) revealed that pneumonia is the most frequent cause of death in PLWD, especially in advanced cases. A critical consideration in nursing care for PLWD who are diagnosed with pneumonia is the occurrence of delirium,^5^ a frequently encountered complication. Delirium presents a complex challenge, demanding heightened vigilance and specialised interventions to ensure optimal patient outcomes. Aliberti et al. (2015) found that about a third of older patients hospitalised with pneumonia showed at least one symptom of delirium. It was shown to be a significant predictor of 1‐year mortality. To further simulate a realistic scenario for nursing interventions, let us consider the case of Margo not only being diagnosed with pneumonia but also exhibiting pronounced signs of delirium. Hence, she possibly suffers from a fluctuating disturbance in attention and orientation to the environment, alteration in other cognitive domains (e.g., delusions) and an altered level of consciousness (American Psychiatric Association 2013).

Shining light on the further progress of dementia, it becomes clear that there is a high probability of change in the balance of pleasure and pain Margo might experience. I will discuss further implications for Margo's well‐being in the next section.

## 4 The Emergence of a New Identity and Further Problems

Assuming Dresser's notion^6^ of advanced dementia transforming Margo 1 into a new Margo 2 at some point in the progression of dementia, we must accept the likelihood that other comorbid medical conditions (and/or the progress of her dementia) could trigger a similar transformation. Other than dementia, delirium's onset occurs rather abruptly and is accompanied by a change in mental status or behaviour (Fong et al. 2015). The duration of delirium among PLWD can vary greatly, ranging from hours and days to a persistent state, and it often leads to fatal outcomes (Fong et al. 2015).

In this essay, I use a variation of the commonly referenced example of the patient called Margo who is living with dementia, where her manifestation of Margo 2 experiences delirium symptoms shortly after the onset of pneumonia. It is highly probable that delirium significantly influences and alters the experiences and behaviours of the affected individual. For instance, Anil Seth, who cared for his delirious mother, described her state as follows: ‘When I find her on the ward she is sitting hunched in the chair, unsmiling, dishevelled, empty‐eyed. She tells me about the people she has seen crawling up the walls, and she cannot remember where she is or why she is here. Her grip on reality, and on who she is, is fading.’ (Seth 2021, 144). Seth's description of his mother is very much like a different numerical identity to the one he used to know.

In line, pneumonia‐induced delirium in Margo 2 could be said to give rise to a new numerical identity, which we will call Margo 3. Due to psychomotor agitation, Margo's behaviour may change, which might result in unsafe actions leading to falls. It may further result in lethargy and psychomotor retardation causing immobility, decreased oral intake and, thereby, malnutrition (Fong et al. 2015). From a hedonistic point of view, this delirious person (Margo 3) has a much worse QoL than the previous one (Margo 2) as there is not only an absence of pleasures and experiential interests but also an increase in unpleasant experiences and pain, turning her life overall bad.

Up to this point, I have shown that PLWD affected by pneumonia are very susceptible to delirium. Because delirium leads to a significant change in cognitive capacity and behaviour, a new numerical person will likely emerge. While Margo 1's critical interests might still persist, Margo 2's experiential interests abruptly vanish.^7^ This raises the question of the moral permissibility of administering treatment to Margo 2—in contrast to Margo 1's AD—based on Margo 2's current experiential interests, ultimately resulting in Margo 2's death^8^ either way. I go on to address this problem and a possible solution in the following sections.

## 5 Death as a Postponed Problem?

**Figure 1:**
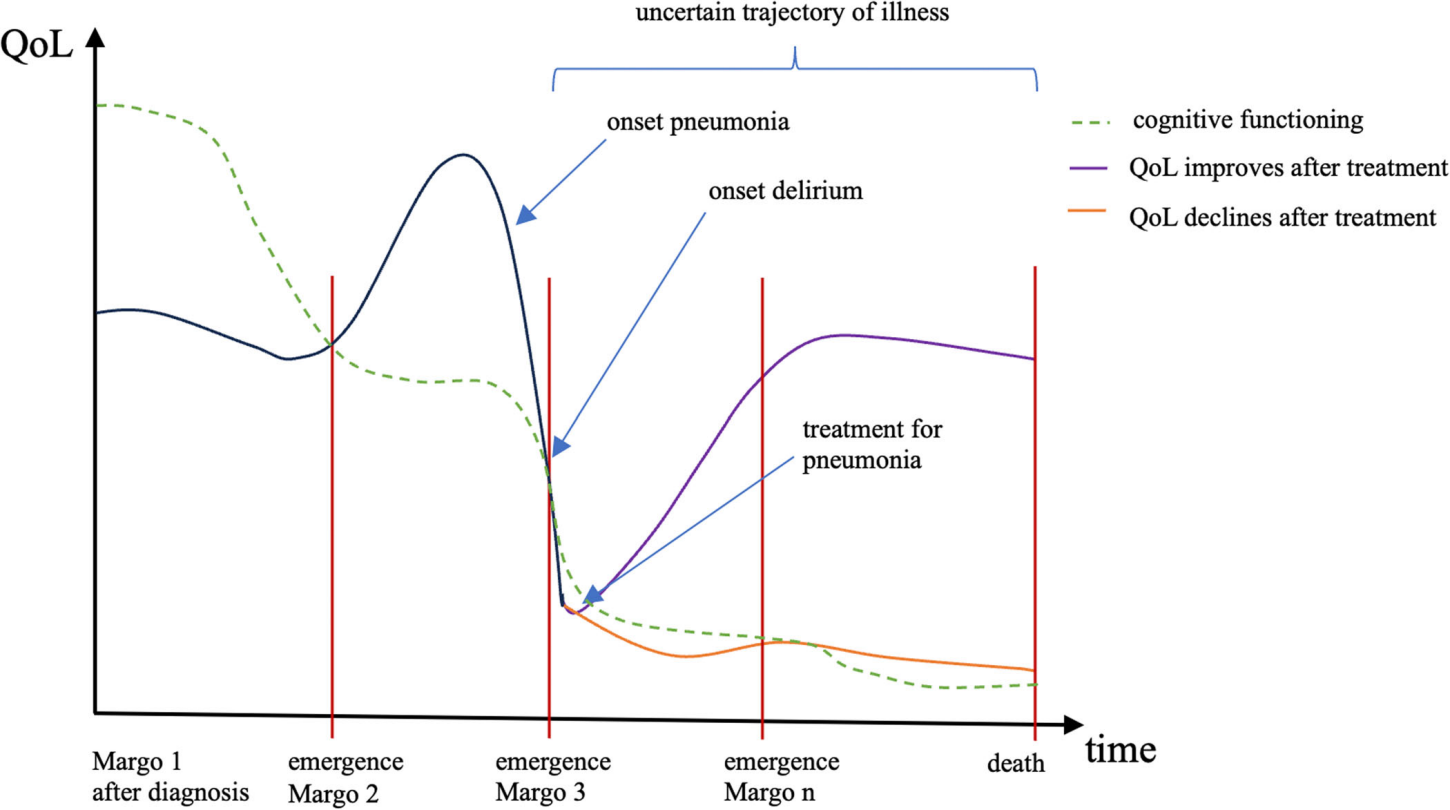
Different trajectories of Margo's QoL and manifestations of different numerical identities over time following potential treatment for pneumonia.

One could argue that by treating Margo 2 and keeping her body alive while Margo 3 emerges, the ethical dilemma was only postponed into the future. It remains unclear whether Margo 3's condition will ever improve. Gross et al. (2012) found that delirium is associated with an increased rate of cognitive deterioration that is maintained for up to 5 years. This suggests that the emergence of Margo 3 amid delirium might signify a trajectory of cognitive deterioration rather than a temporary setback, resulting in future manifestations of Margo. Thus, the decision to prolong Margo's life amidst such complexities raises profound ethical questions about the QoL and the dignity of the individual. Given the relentless trajectory of cognitive decline and the consequent loss of psychological connections, it is increasingly likely that we will witness additional manifestations of Margo's evolving state until her body finally dies. Figure 1 shows possible trajectories of Margo's QoL in the course of her illness.

In situations where it remains uncertain whether Margo's well‐being,^9^ in any of her future manifestations, will be enhanced by life‐sustaining interventions, her critical interests ought to take precedence over her experiential ones. As neither Margo 2 nor Margo 3 are able to assess possible risks, side effects or benefits of treatment, Margo 1's AD should be taken into account. Subjecting any future manifestation of Margo and her body to possible suffering is morally impermissible if the outcome of life‐sustaining measures on her QoL is not predictable, as shown in Figure 1. The case of Margo 3 is such that even healthcare professionals cannot predict the impact of treatment on her overall well‐being, given the uncertainty of whether delirious symptoms would persist or not.

As a consequence, it seems unjustifiable to keep Margo 3 alive based on Margo 2's past QoL and interests, rather than choosing to withhold treatment, causing Margo 2's death. Reflecting on Margo 1's AD, she might have intended to preclude the eventuality of having to suffer from the potential consequences of comorbidities.^10^ In weighing the decision of whether it constitutes an act of beneficence to sustain Margo in one of her evolving states through treatment or to honour Margo 1's expressed wishes as outlined in her AD, we might look at Bester (2020) approach for decision‐making in medical encounters. Bester (2020) introduces a set of distinct criteria pertaining to the patient's interests and overall well‐being. Bester argues that in cases involving critically‐ill patients, such as Margo, when a patient chooses comfort care over curative treatment, the concept of benefit undergoes a significant shift. Here, efforts aimed at curing the underlying condition^11^ are no longer viewed as beneficial; rather, they may be perceived as burdensome to the patient. Instead, the focus shifts towards benefits that alleviate distressing symptoms and ensure a dignified end‐of‐life experience. This redefinition of benefit persists even in scenarios where potentially curative treatments, such as antibiotics for pneumonia, are available. It's crucial to note that such treatments may address Margo's acute illness, they do not offer a cure for chronic conditions like dementia or delirium, which are central to Margo's case.

Expanding upon his argument, Bester (2020) advances a novel conception of well‐being tailored for medical practice. This revised framework encompasses the criterion of objective functioning (1), which encompasses aspects such as pain management and cognitive function. Additionally, Bester emphasises the importance of evaluating the patient's ability to achieve personal goals and fulfil their deeply held values^12^ (2) as integral components of their overall well‐being. Each of Criteria (1) and (2) has the potential to establish conditional *prima facie* obligations for the clinician, and either one can override or alter the *prima facie* obligations generated by the other criterion.

Margo 3's current delirious state suggests a heightened probability of ongoing cognitive decline, potentially exacerbating her objective impairment, as illustrated in Figure 1. The uncertainty surrounding her ability to communicate complicates decision‐making. Bester's criterion of personal goals and values implies enduring moral foundations, which resonate deeply with Margo 1's beliefs and life goals, since Margo 3 is incapable of forming critical interests. Therefore, Margo 1's AD carries significant moral weight, guiding decisions to honour her autonomy and dignity amid cognitive decline. Upholding Margo 1's expressed wishes navigates the complexities of her care journey. Furthermore, empirical evidence indicates that her cognitive decline, coupled with symptoms like pain and anxiety, may significantly diminish Margo 3's QoL (Hendriks et al. 2014), also impacting her experiential well‐being. This perspective is consistent with the assertion that continuing Margo 3's treatment based on Margo 2's past condition is unjustified and may cause her unnecessary suffering and reduce her overall QoL. Reflecting on Margo 1's AD highlights the importance of respecting her expressed wishes, which may have been to avoid potential comorbidity consequences. Administering treatment under these circumstances does not constitute a beneficent act, as neither version of Margo benefits from it.

In this section, I argued against disregarding Margo 1's AD, highlighting the potential prolongation of her suffering and the inevitable decline in her QoL as her cognitive and physiological functions deteriorate. This perspective aligns with Bester's framework for evaluating well‐being, which emphasises honouring the patient's expressed values (i.e., critical interests). While Margo's physical condition remains shrouded in uncertainty, one stark certainty looms over the situation: the inevitability of her body's eventual demise, regardless of the form or manifestation it currently harbours. It seems that the treatment of Margo 2's pneumonia has only bought time until the inevitable death of Margo's body, causing unnecessary suffering to Margo 3 (or possibly future manifestations) as its current inhabitant. I will therefore explore the concept of Margo 1's prior autonomy, based on her inherent ownership of her body. This exploration will illuminate the ethical implications surrounding Margo's autonomy and the rights she retains over her own bodily integrity, despite the evolving complexities of her condition.

## 6 Self‐Ownership and Beneficence

As already outlined, the principle of beneficence should not solely prioritise the physical well‐being of the patient, but also her interests (i.e., her moral values) (Bester 2020; Kinsinger 2009). Dworkin argues that following a patient's AD ought to be considered a beneficial act, criticising failure to comply with Margo 1's AD as lacking compassion and unjustified paternalism (Dworkin 1993). Dresser, however, suggests considering beneficence in terms of Margo 2's current QoL, emphasising the caregivers' perspective on Margo's overall well‐being as a more appropriate basis for medical decision‐making than solely relying on Margo 1's AD (Dresser 1995). Nevertheless, given Margo 3's delirium and related symptoms, a hedonistic perspective suggests that her decreased QoL and limited experiential interests indicate poor well‐being.

All manifestations of Margo share the fact that they reside in the same body. We might assume that Margo 1 has self‐ownership over the body as she lived in it first. According to Furberg (2012), there may be a conflict between Margo 1's right to self‐ownership, which is a quasi‐property right over her body, and Margo 2's (as well as Margo 3's) right to self‐determination. Buchanan and Brock (1991, 166) suggest that the right to self‐ownership could be justifiably overridden ‘to secure some very important good or avoid some important harm.’ Furberg (2012) concludes that the issue of personal identity does not necessarily diminish the moral authority of AD in PLWD, as Dresser argues—it only does in specific circumstances. Given the many uncertainties regarding Margo's future well‐being and her current poor QoL, and that Margo 3's right to self‐determination is limited due to her cognitive incapacity, Margo 1's right to self‐ownership cannot easily be overridden. Hence, Margo 1's critical interests in her body, as expressed in her AD, take precedence over the present experiential interests of Margo 2 and 3.

So far, I have argued that we have three reasons to follow Margo 1's AD: (1) complications like delirium will probably cause a new manifestation of Margo to emerge, and (2) this manifestation (Margo 3) will very likely be low in experiential interests and have a poor QoL. (3) Thus, as the future course of Margo's illness is very uncertain, her critical interests as reflected by her AD and her right to self‐ownership over her body, ought to have moral authority over new numerical identities. I will now discuss some objections that may be raised against my argument.

## 7 Objections

Dresser might object that letting Margo 3 die does not increase her well‐being. Assuming a deprivation account of death, this would deprive her from life's goods (i.e., experiential interests). As stated above, it is highly questionable whether or not Margo will recover from pneumonia and delirium, making death inevitable. It also highly depends on whether Margo 3's current overall well‐being is good or poor. Considering the described case of a delirious Margo who might be experiencing delusions, showing unsafe behaviours and is suffering from physical disabilities, her overall well‐being might very likely be poor. In this case, death could count as an increase in overall well‐being.

What if Margo 2 contracts pneumonia that is easily treatable and does not develop delirious symptoms, for example, because her overall physical health is still relatively intact though her cognitive functioning has severely declined due to dementia? It seems that my argument does not apply to this particular case. However, research indicates a prevalent occurrence of PLWD in nursing facilities who endure a considerable symptom burden, notably marked by high levels of pain (Helvik et al. 2022; Hendriks et al. 2014). Moreover, these individuals often receive poorer quality end‐of‐life care compared to patients with cancer (Martinsson, Lundström, and Sundelöf 2018). Hence, even in scenarios where medical interventions can address acute conditions, the overall well‐being of PLWD is often compromised due to the cumulative effects of dementia‐related symptoms and systemic deficiencies in health care for this population. Thus, while the scenario of easily treatable pneumonia may initially appear to deviate from the argument presented, the broader context of the experiences of PLWD highlights the continued relevance of considering critical interests and respecting ADs in ethical decision making.

According to Huang, Cong, and Wang (2022), medical decisions should not be solely reliant on either present QoL assessments or precedence of autonomy. They advocate for physicians to aid individuals diagnosed with Alzheimer's in crafting ADs during the early stages of the disease when they are still competent. This proactive approach aims to foster a collaborative decision‐making process. Implementing ADs at this juncture may also enhance the receptiveness of PLWD to engage in the creation of such directives.^13^ Their methodology shares similarities with Bester (2020) approach. I agree that ADs should be the outcome of an informative communication process, wherein individuals affected should be thoroughly educated about the disease and its potential complications. Given that the present jurisdiction in Germany does not mandate the involvement of a healthcare professional, this is of particular concern. As mentioned earlier, QoL encompasses various aspects. Therefore, even if pneumonia can be effectively treated and physical QoL is satisfactory, overall well‐being may still be poor (Huang, Cong, and Wang 2022).

One might also object that following Margo 1's AD is equal to granting her posthumous wishes. On the other hand, ignoring her AD could cause harm according to the posthumous harm theory. The no‐subject objection claims that there simply is no one who could be harmed after death. However, in Margo 1's case, we may distinguish between her vanished personal identity and her living body, which remains physically connected to her past. Nevertheless, even if we agree that following her AD is equivalent to fulfilling her posthumous wishes, Persad (2019) suggests that an individual in the pre‐dementia stage should have posthumous rights over their body. He bases his argument on historical embodiment which can override moral claims based on current embodiment. Persad draws an analogy to posthumous pregnancy and suggests ‘the predementia individual may similarly refuse to provide bodily support to the post‐dementia individual even when this will lead to the post‐dementia individual's death’ (Persad 2019).

In this section, I showed that Dresser might object, arguing that allowing Margo 3 to die may not necessarily improve her well‐being. However, given Margo's current condition and uncertainties regarding her recovery, her overall well‐being may already be poor, making death potentially preferable. Additionally, research indicates that PLWD often endure significant symptom burdens and receive poorer quality end‐of‐life care. PLWD ought to be involved in creating ADs early in the disease's progression to improve decision‐making. Despite objections, Persad argues for posthumous rights over the body for individuals in the pre‐dementia stage, drawing parallels to historical embodiment and posthumous pregnancy.

## 8 Conclusion

In this paper, I criticise Dresser's argument on the lack of moral authority of ADs for PLWD based on Parfit's personal identity account. Therefore, I used a variation of the commonly referenced example of demented Margo, where Margo 2 experiences delirium symptoms shortly after the onset of pneumonia. I limit my argument to only consider critical interests regarding one's body. I argue that this common comorbidity of dementia might lead to a new numerical identity emerging that will experience an abrupt decrease in experiential interests and QoL due to psychological and physical complications. As it is uncertain whether Margo will ever recover from this state, Margo 1's critical interests as reflected by her AD as well as her right to self‐ownership over her body ought to take precedence over demented Margo's experiential interests. Therefore, Margo 1's AD possesses moral authority.

By critically examining arguments put forth by scholars such as Dworkin, Dresser, and Bester, I have elucidated the nuanced considerations that must be weighed when making decisions about the care of PLWD. While acknowledging the importance of recognising the experiential interests and current QoL of patients, I have underscored the paramount importance of honouring the critical interests expressed in ADs. These critical interests serve as a guiding principle for ethical decision‐making, ensuring that care aligns with the deeply held values and preferences of patients, even in the face of evolving health conditions with uncertain trajectories.

Moving forward, it is imperative that healthcare professionals continue to engage in informed discussions with patients and their families about the creation and utilisation of ADs in the early stages of dementia. By doing so, we can empower individuals to assert their autonomy and dignity throughout the progression of their illness. Ultimately, by prioritising the critical interests expressed in ADs and adopting a patient‐centred approach to care, we can navigate the complexities of dementia care with compassion and respect for the individual's values and wishes.

## Ethics Statement

The author has nothing to report.

## Consent

The author has nothing to report.

## Conflicts of Interest

The author declares no conflicts of interest.

## Data Availability

The author has nothing to report.

## Endnotes

1 In Germany, ADs are legally binding as long as the affected person is legally capable while signing the AD. Therefore, physicians or notaries can be consulted but do not have to take part in the process.

2 Given that assisted dying is currently a highly debated topic in the UK, the organisation ‘Dignity in Dying’ has established the following key elements to provide a definition of this practice: (1) control over where we die, who is present and our treatment options; (2) access to expert information on our options, good quality end‐of‐life care; and (3) control over how we die, our symptoms, pain relief and planning our own death. (Campaign for Dignity in Dying 2023)

3 Parfit describes psychological connectedness as the ‘holding of particular direct psychological connections.’ He defines psychological continuity as the ‘holding of overlapping chains of strong connectedness’ (Parfit 1984, 206–207).

4 I assume, contra Walsh (2020), that Margo 2 is not capable of developing new or different critical interests because of her declining cognitive capacities.

5 I am referring to delirium as a condition unique to a geriatric population of patients, not delirium tremens due to alcohol use. The interrelation between delirium and dementia is still poorly understood. Evidence exists for multifactorial causes for delirium (Fong et al. 2015).

6 As Dworkin's argument is consistent with surviving interests, I will assume that he accepts that there is a break in personality.

7 Her experiential interests may not disappear entirely, but they will likely decrease significantly. This will create an imbalance between pleasure and pain and, therefore, a poorer QoL.

8 If we posit that Margo 3's emergence signifies the end of Margo 2's existence in terms of her numerical identity, rather than her physical body.

9 While there remains a possibility that Margo 3's QoL may improve with antibiotic treatment, empirical evidence indicates otherwise. However, in order to emphasise the uncertainty of the trajectory, the possibility of an increase has been included in this figure.

10 Dresser (1995) presumes that Margo 1 could not predict her future QoL as a PLWD and, as a result, misconstrued her future state due to the stigma attached to dementia. We might as well hypothesise that Margo 1 was aware of the fact that her demented self might lead a happy and content life but preferred her life to be terminated upon contracting a life‐threatening disease to prevent her from any further suffering.

11 While advanced dementia and dementia in general currently lack a cure, it is important to note that pneumonia, here the underlying condition leading to delirium, can be treated with antibiotics. However, it is crucial to recognise that curing pneumonia does not necessarily equate to curing delirium.

12 Bester's approach (2020) initially draws from various theories of QoL, including the desire‐fulfilment theory and the objective list theory. Within this framework, desires can be classified into experiential, such as craving a specific food, or critical, for example, involving advocacy for certain moral principles. The objective list theory tends to align more closely with critical interests. However, Bester's approach appears compatible with the hedonist perspective, which is why it is discussed in this essay. Additionally, Dresser herself noted that many individuals do not distinguish between experiential and critical interests, but rather evaluate QoL based on whether it is perceived as good or poor.

13 According to Dresser (1995), the creation of ADs is a practice that is followed by only a small percentage of PLWD. I believe that this can be attributed to various factors, such as the fear of confronting impending death or a lack of awareness and understanding about ADs. However, it would be incorrect to interpret this low participation rate as solid evidence of the insignificance of ADs.

## References

[nup70016-bib-0001] Aliberti, S. , G. Bellelli , M. Belotti , et al. 2015. “Delirium Symptoms During Hospitalization Predict Long‐Term Mortality in Patients With Severe Pneumonia.” Aging Clinical and Experimental Research 27: 523–531. 10.1007/s40520-014-0297-9. 25556562

[nup70016-bib-0002] American Psychiatric Association . 2013. Diagnostic and Statistical Manual of Mental Disorders (DSM‐5‐TM), 5th ed. Washington, D.C.: American Psychiatric Association.

[nup70016-bib-0003] Bester, J. C. 2020. “Beneficence, Interests, and Wellbeing in Medicine: What It Means to Provide Benefit to Patients.” American Journal of Bioethics 20, no. 3: 53–62. 10.1080/15265161.2020.1714793 32105204

[nup70016-bib-1001] Buchanan, A. , and D. W. Brock . 1991. “Deciding for Others.” Philosophical Quarterly 41, no. 162: 118–119.

[nup70016-bib-0004] Bunn, F. , A.‐M. Burn , C. Goodman , et al. 2014. “Comorbidity and Dementia: A Scoping Review of the Literature.” BMC Medicine 12, no. 1: 192. 10.1186/s12916-014-0192-4. 25358236 PMC4229610

[nup70016-bib-0005] Campaign for Dignity in Dying . 2023. Accessed June 26, 2023. https://www.dignityindying.org.uk/.

[nup70016-bib-0006] Cerejeira, J. , L. Lagarto , and E. B. Mukaetova‐Ladinska . 2012. “Behavioral and Psychological Symptoms of Dementia.” Frontiers in Neurology 3: 73. 10.3389/fneur.2012.00073. 22586419 PMC3345875

[nup70016-bib-0007] Degerskär, A. N. W. , and E. M. Englund . 2020. “Cause of Death in Autopsy‐Confirmed Dementia Disorders.” European Journal of Neurology 27, no. 12: 2415–2421. 10.1111/ene.14450. 32692883

[nup70016-bib-0008] Dresser, R. 1995. “Dworkin on Dementia: Elegant Theory, Questionable Policy.” Hastings Center Report 25, no. 6: 32–38. 8609018

[nup70016-bib-0010] Dworkin, R. 1993. Life's Dominion: An Argument About Abortion and Euthanasia. New York: HarperCollins Publishers.

[nup70016-bib-0011] Fischer, J. , C. Roßmeier , and J. Hartmann , et al. 2023. “Inappropriate Involvement? Presenting Empirical Insight Into the Preparation Phase of Advance Directives of Persons Living With Dementia Under German Legislation.” Journal of Aging and Social Policy 36, no. 5: 841–856. 10.1080/08959420.2023.2182565. 36814064

[nup70016-bib-0012] Fong, T. G. , D. Davis , M. E. Growdon , A. Albuquerque , and S. K. Inouye . 2015. “The Interface Between Delirium and Dementia in Elderly Adults.” Lancet Neurology 14, no. 8: 823–832. 10.1016/S1474-4422(15)00101-5. 26139023 PMC4535349

[nup70016-bib-0013] Froelich, L. , A. Lladó , R. K. Khandker , et al. 2021. “Quality of Life and Caregiver Burden of Alzheimer's Disease Among Community Dwelling Patients in Europe: Variation by Disease Severity and Progression.” Journal of Alzheimer's Disease Reports 5, no. 1: 791–804. 10.3233/ADR-210025. PMC8609484 34870105

[nup70016-bib-0014] Furberg, E. 2012. “Advance Directives and Personal Identity: What Is the Problem?” Journal of Medicine and Philosophy 37, no. 1: 60–73. 10.1093/jmp/jhr055. 22190599

[nup70016-bib-0015] German Alzheimer Society, e.V . 2023, Mai 13. Die Häufigkeit von Demenzerkrankungen. https://www.deutsche-alzheimer.de/fileadmin/Alz/pdf/factsheets/infoblatt1_haeufigkeit_demenzerkrankungen_dalzg.pdf.

[nup70016-bib-0016] Gross, A. L. , R. N. Jones , D. A. Habtemariam , et al. 2012. “Delirium and Long‐Term Cognitive Trajectory Among Persons With Dementia.” Archives of Internal Medicine 172, no. 17: 1324–1331. 23403619 10.1001/archinternmed.2012.3203 PMC3740440

[nup70016-bib-0017] Helvik, A.‐S. , S. Bergh , J. Šaltytė Benth , G. Selbaek , B. S. Husebo , and K. Tevik . 2022. “Pain in Nursing Home Residents With Dementia and Its Association to Quality of Life.” Aging and Mental Health 26, no. 9: 1787–1797. 10.1080/13607863.2021.1947968. 34251936

[nup70016-bib-0018] Hendriks, S. A. , M. Smalbrugge , C. M. P. M. Hertogh , and J. T. van der Steen . 2014. “Dying With Dementia: Symptoms, Treatment, and Quality of Life in the Last Week of Life.” Journal of Pain and Symptom Management 47, no. 4: 710–720. 23916680 10.1016/j.jpainsymman.2013.05.015

[nup70016-bib-0019] Huang, Y. , Y. Cong , and Z. Wang . 2022. “Rethinking the Precedent Autonomy, Current Minimal Autonomy, and Current Well‐Being in Medical Decisions for Persons With Dementia.” Journal of Bioethical Inquiry 19, no. 1: 163–175. 35015243 10.1007/s11673-021-10159-3

[nup70016-bib-1002] Kaufmann, B. 2015. Patientenverfügungen zwischen Selbstbestimmung und staatlicher Fürsorge–Mehr Patientenautonomie durch das 3. BtÄndG? Würzburg University Press.

[nup70016-bib-0020] Kinsinger, F. S. 2009. “Beneficence and the Professional's Moral Imperative.” Journal of Chiropractic Humanities 16, no. 1: 44–46. 10.1016/j.echu.2010.02.006. 22693466 PMC3342811

[nup70016-bib-0021] Martinsson, L. , S. Lundström , and J. Sundelöf . 2018. “Quality of End‐Of‐Life Care in Patients With Dementia Compared to Patients With Cancer: A Population‐Based Register Study.” PLoS One 13, no. 7: e0201051. 10.1371/journal.pone.0201051. 30059515 PMC6066197

[nup70016-bib-0022] Parfit, D. 1984. Reasons and Persons. Oxford: OUP Oxford.

[nup70016-bib-0023] Persad, G. 2019. “Authority Without Identity: Defending Advance Directives via Posthumous Rights Over One's Body.” Journal of Medical Ethics 45, no. 4: 249–256. 30580321 10.1136/medethics-2018-104971

[nup70016-bib-0024] Seth, A. 2021. Being You: A New Science of Consciousness. London: Faber & Faber.

[nup70016-bib-0025] Vollmann, J. 2001. “Advance Directives in Patients With Alzheimer's Disease; Ethical and Clinical Considerations.” Medicine, Health Care, and Philosophy 4: 161–167. 11547502 10.1023/a:1011491100267

[nup70016-bib-0026] Walsh, E. 2020. “Cognitive Transformation, Dementia, and the Moral Weight of Advance Directives.” American Journal of Bioethics 20, no. 8: 54–64. 10.1080/15265161.2020.1781955 32757910

